# CO_2_ Fixation with Epoxides under Mild Conditions with a Cooperative Metal Corrole/Quaternary Ammonium Salt Catalyst System

**DOI:** 10.1002/asia.201700354

**Published:** 2017-04-20

**Authors:** Maximilian Tiffner, Sabrina Gonglach, Michael Haas, Wolfgang Schöfberger, Mario Waser

**Affiliations:** ^1^ Institute of Organic Chemistry Johannes Kepler University Linz Altenberger Straße 69 4040 Linz Austria

**Keywords:** carbon dioxide fixation, cooperative catalysis, corroles, quaternary ammonium salts, sustainable chemistry

## Abstract

The cooperative catalytic activity of several metal corrole complexes in combination with tetrabutyl‐ammonium bromide (TBAB) has been investigated for the reaction of epoxides with CO_2_ leading to cyclic carbonates. It was found that the use of just 0.05 mol % of a manganese(III)corrole with 2 mol % TBAB exhibits excellent catalytic activity under an atmosphere of CO_2_.

Carbon dioxide (CO_2_) is available in almost infinite amounts in our atmosphere and oceans, but its utilization as feedstock for the chemical industry is often prevented by its thermodynamic stability. Nevertheless, significant progress has been made in the utilization of CO_2_ as a simple C1 synthon for organic synthesis over the last years.^1^ However, only a few large‐scale industrial processes utilizing CO_2_ as a simple feedstock for organic reactions have been achieved so far, such as the production of urea, methanol, salicylic acid, or cyclic carbonates.

Dioxolanones are useful intermediates to synthesize vicinal diols,^2^ which are industrially useful monomers, polymers, surfactants, plasticizers, cross‐linking agents, curing agents, and solvents, to name a few applications only.^3^ The synthesis of organic carbonates by catalytic insertion of carbon dioxide into a carbon–oxygen bond of an epoxide usually requires the use of high temperature and pressure. Various catalysts for this reaction have been developed, including metal complexes^4^, ^5^, ^6^, ^7^, ^8^, ^9^, ^10^ (e.g., metal–salen complexes and metalloporphyrins) and organocatalysts (e.g., quaternary onium salts or N‐heterocyclic carbenes).^11^ Among these catalysts, metalloporphyrins have shown relatively high catalytic activity under CO_2_ autoclave conditions (>5 bar) and at elevated temperatures (usually >100 °C).^4^, ^8^, ^9^, ^10^ In contrast to porphyrin‐based systems, the closely related corrole macrocycle can stabilize metal ions in higher oxidation states,^12^, ^13^, ^14^, ^15^, ^16^ making them unique reagents with extraordinary catalytic properties.^17^, ^18^ In this study, we focused on manganese, iron, cobalt, copper, antimony and bismuth 5,10,15‐tris(pentafluorophenyl) corrole (MTpFPC) complexes **1 a**–**f** (Figure 1) for CO_2_ fixation reactions. The center metal ions are complexed as Bi^III^,^13^ Co^IV^,^14^ Cu^II^,^15^ Fe^IV^,^14^, ^16^ and Mn^III^,^14^ accordingly, and the three C_6_F_5_‐groups in the *meso* positions 5, 10, and 15 of the macrocycle withdraw electron density from the 18‐π‐electron system. A consequence of this effect is the improved stability of such high‐valent metal corroles. Nozaki and co‐workers recently reported the use of Fe corroles and bis(triphenylphosphine)iminium chloride as an additive for the copolymerization of epoxides with CO_2_ under high pressure conditions.^18^ Interestingly, in this case study, the formation of the cyclic carbonates was more or less totally suppressed. Based on our recent interest in the use of metal corroles as catalysts17a and the recent progress in the use of cooperative CO_2_‐fixation catalyst systems based on metal complexes in combination with simple nucleophilic halide sources such as tetrabutylammonium bromide (TBAB),^5^ we reasoned that the use of alternative metal‐based corroles together with TBAB may result in a very powerful cooperative catalyst system for the CO_2_ fixation with epoxides. Such a catalyst system may even operate under an atmospheric pressure of CO_2_ at low temperature. Because of this lower CO_2_ pressure, we argued that this synergistic catalyst combination may allow us to selectively access cyclic carbonates instead of polymerization products, which would thus result in a highly complementary approach to Nozaki's impressive polymerization protocol^18^ by relying on a similar corrole system.

**Figure 1:**
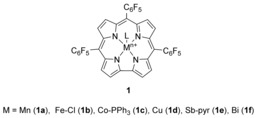
Structures of metal corrole complexes **1 a**–**f** used in this work.

Table 1 gives an overview of the most significant results obtained in a detailed screening of different metal corroles **1** in combination with TBAB as a cheap nucleophilic organic halide source for the solvent‐free CO_2_ fixation of styrene oxide (**2 a**) under an atmosphere of CO_2_ (using a balloon). As expected, only the synergistic combination of corroles **1** and TBAB allows for a reasonable conversion within a relatively short reaction time (at slightly elevated temperatures). In contrast, the absence of either **1** or TBAB resulted in no or only very slow formation of **3 a** only (entries 1–4). Testing of the different metal corroles **1 a**–**f** next showed that Mn‐ and Fe‐based ones clearly outperformed the other metal complexes tested herein (entries 1 and 5–9). Owing to the superior catalytic performance of Mn‐corrole **1 a** we further fine‐tuned the reaction conditions with this system (entries 10–13). Hereby, it was found that reducing the catalyst loading below 0.01 mol % **1 a** resulted in a reduced conversion rate (entries 10 and 11). On the other hand, carrying out the reaction with 0.05 mol % **1 a** and 2 mol % TBAB (entry 12) leads to a higher conversion, and a slightly longer reaction time of 8 h results in almost full conversion of **2 a** under relatively mild conditions with low Mn‐corrole loadings (entry 13).

**Table 1:** Identification of the optimum catalyst system for the CO_2_ fixation of epoxide **2 a**. 
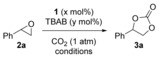

Entry^[a]^	**1** [mol %]	TBAB [mol %]	*T* [°C]	*t* [h]	Conv.^[b]^ [%]
1	Mn **1 a** (0.05 %)	1 %	60	4	48
2	–	1 %	60	4	9
3	Mn **1 a** (0.05 %)	–	60	4	n.r.
4	Mn **1 a** (0.05 %)	1 %	25	4	8
5	Fe **1 b** (0.05 %)	1 %	60	4	34
6	Co **1 c** (0.05 %)	1 %	60	4	13
7	Cu **1 d** (0.05 %)	1 %	60	4	13
8	Sb **1 e** (0.05 %)	1 %	60	4	12
9	Bi **1 f** (0.05 %)	1 %	60	4	15
10	Mn **1 a** (0.01 %)	1 %	60	4	29
11	Mn **1 a** (0.003 %)	1 %	60	4	15
12	Mn **1 a** (0.05 %)	2 %	60	4	60^[c]^
13	Mn **1 a** (0.05 %)	2 %	60	8	>95^[d]^

[a] 4 mmol scale (neat); [b] determined by ^1^H NMR of the reaction mixture; [c] less than 15 % conversion in the absence of **1 a**; [d] the product could be quantitatively isolated after filtration over a short plug of silica.^19^ n.r.=no reaction.

Having identified the best‐suited cooperative catalyst combination and reaction conditions for the solvent‐free CO_2_‐fixation of epoxide **2 a**, we next investigated the scope of this protocol by using other simple epoxides **2** (Table 2). Most of the epoxides reacted at a similar rate to the parent styreneoxide **2 a** at 60 °C. Only the diphenylmethylether‐based starting material (entry 6) and epichlorhydrine (entry 7) showed a slightly slower conversion of less than 90 % under standard conditions. In contrast, some aliphatic epoxides even showed good conversion at room temperature (entries 10–12), thus proving the generality of this method for the CO_2_ fixation with epoxides **2**.

**Table 2:** Application scope. 
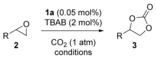

Entry^[a]^	R	*T* [°C]	*t* [h]	Conv.^[b]^ [%]	Yield^[c]^ [%]	TON^[d]^	TOF^[d]^ [h^−1^]
1	Ph	60	8	>95	94	1880	235
2	Ph	25	8	25	24	480	60
3	4‐Cl‐C_6_H_4_	60	8	>98	96	1920	240
4	4‐F‐C_6_H_4_	60	8	>98	98	1960	245
5	PhOCH_2_	60	8	>95	94	1880	235
6	Ph_2_CHOCH_2_	60	8	71	70	1400	175
7	ClCH_2_	60	8	89	88	1760	220
8	vinyl	60	8	>98	93	1860	232
9	but‐3‐enyl	60	8	>98	98	1960	245
10	but‐3‐enyl	25	20	>98	98	1960	98
11	Me	25	8	57	55	1100	137
12	Me	25	20	>95	88	1760	88

[a] 4 mmol scale (neat); [b] judged by ^1^H NMR of the reaction mixture; [c] isolated yield after filtration over a short plug of silica; [d] based on **1 a**. TON=turnover number; TOF=turnover frequency.

The synergistic effect of an organic nucleophilic halide source and a Lewis acidic metal complex for these CO_2_‐fixation reactions has been the subject of detailed recent mechanistic studies.^5^, ^7^, ^8^ For example the groups of North et al. and Ren and Lu have done systematic investigations of salen‐ and salphen‐based systems,^5^, ^7^ while Hasegawa et al. have carried out very detailed DFT investigations for porphyrin‐based catalyst systems at elevated CO_2_ pressure.^8^ Based on these comprehensive studies we propose that the herein‐reported Mn‐corrole **1 a**/TBAB system operates through an analogous mechanism (Scheme 1). To corroborate the mechanism, we performed DFT calculations of the three main proposed intermediates **A**–**C**. The corrole macrocycle exhibits a dome‐shaped structure after coordination with the ring‐opened substrate (intermediate **A**) and the manganese atom lies slightly above the plane defined by the four nitrogen atoms of the corrole ring. The Mn atom is coordinated by the four nitrogen atoms and axially by the oxygen atom of the ring‐opened epoxide. After the insertion reaction of CO_2_, the axial pyramidal conformation is distorted (see structure **B**). Herein, one O atom originating from CO_2_ is axially coordinating with a distance of 1.8 Å to the manganese ion, and the other oxygen atom is 2.86 Å apart from the Br methylene group and can easily perform, in the final step, the ring‐closure to the cyclic carbonate **3**.

**Scheme 1:**
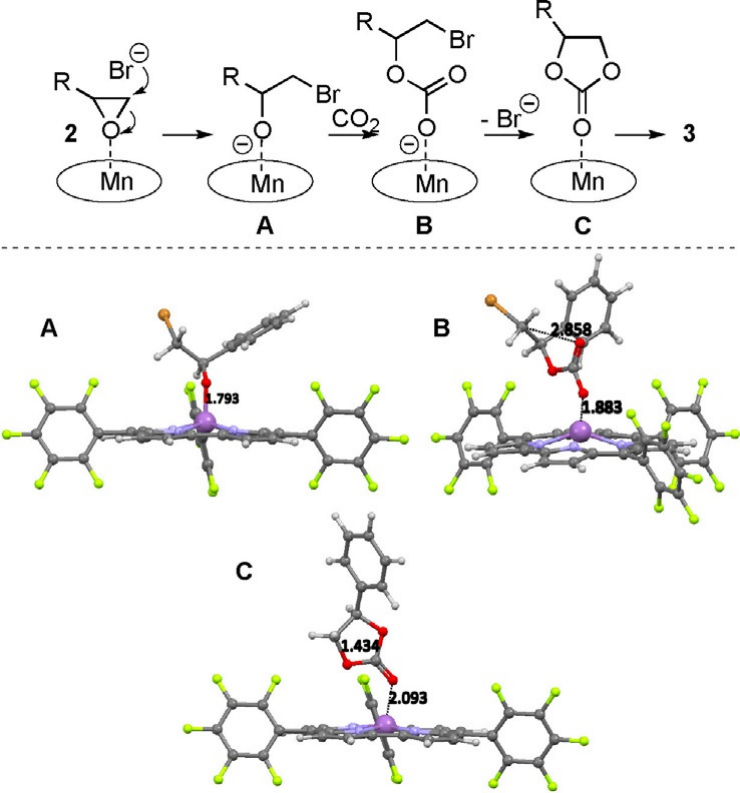
Proposed synergistic catalysis mode for the Mn‐corrole **1 a**‐ and TBAB‐catalyzed CO_2_ fixation with epoxides (based on recent studies),^7^, ^8^ and calculated molecular structures of the proposed intermediates **A**–**C**.

To obtain further mechanistic details, we investigated the time course UV/Vis spectral changes occurring to the catalyst **1 a** during the reaction. The typical UV/Vis absorption spectrum of **1 a** and propylene oxide (Figure 2, solid black line, 1) changes significantly after addition of TBAB under a CO_2_ atmosphere (dotted green line, 2). While the Soret band maximum at 410–420 nm remained unaffected, a strong increase of the absorption band and a hypsochromic shift from 485 to 472 nm was immediately observed (Figure 2, transition 1→2). The latter absorption band is known to be very sensitive towards axial ligation and the change in absorption wavelength can be attributed to the binding of an axially ligated oxygen atom either as alkoxide intermediate **A** and/or carbonate intermediate **B** (compare with Scheme 1).^20^, ^21^ To get further information regarding the expected intermediates (Scheme 1) and the apparent suggestion that the electronic spectra presented in Figure 2 reflect them, we compared the spectral changes upon coordination of simple model compounds to corrole **1 a** (i.e., an alkoxide for intermediate **A**, a carboxylate for intermediate **B**, Figures S1 and S2). The hereby obtained UV/Vis spectra are identical to the UV/Vis spectrum of **1 a** in the presence of propylene oxide, TBAB and CO_2_ (dotted green line, Figure 2) and clearly support the presence of an axially ligated oxygen.^20^, ^21^ However, no noticeable differences between the spectra obtained upon coordination of an alkoxide or a carboxylate to **1 a** could be detected, still making it impossible to unambiguously assign the illustrated UV/Vis spectrum in Figure 2 (dotted green line, 2) to either intermediate **A** or **B**.

**Figure 2:**
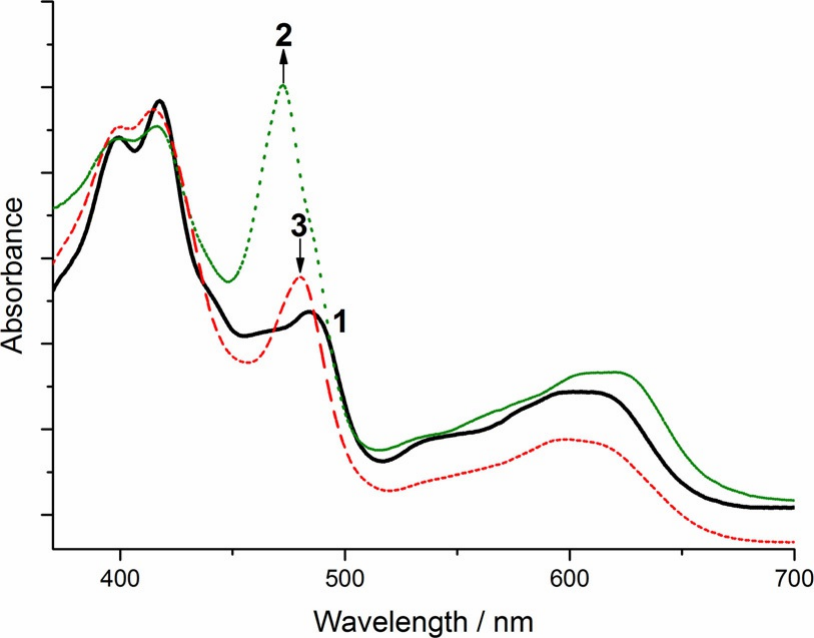
UV/Vis absorption spectra of **1 a** during the CO_2_ fixation reaction of propylene oxide to 4‐methyl‐1,3‐dioxolan‐2‐one. Solid black line: **1 a** in propylene oxide at 20 °C; dotted green line: after addition of propylene oxide, TBAB and CO_2_ bubbling; dashed red line: after full conversion of propylene oxide to cyclic carbonate **3**.

Finally, after full conversion of propylene oxide to carbonate **3** the UV/Vis absorption spectrum (Figure 2, dashed red line, 3) changes back, comparably to the one observed in the beginning for the non‐reacted catalyst species **1 a** (Figure S3).

After recycling the manganese corrole species (column chromatography of the reaction mixtures first with heptanes/EtOAc=10:1–3:1 to isolate the carbonates and then with heptanes/EtOAc=1:1 to elute the catalyst), analysis of ESI‐MS and ^19^F NMR spectra (Figures S4 and S5) revealed that the manganese corrole remained intact and was reusable for further transformations.

To conclude, we have identified that TBAB/Mn^III^corrole **1 a** is the best‐suited cooperative catalyst combination so far. Owing to the superior catalytic performance of Mn‐corrole **1 a** we further fine‐tuned the reaction conditions with this system. Hereby, it was found that reducing the catalyst loading below 0.01 mol % **1 a** resulted in a reduced conversion rate. On the other hand, carrying out the reaction with 0.05 mol % **1 a** and 2 mol % TBAB led to an increased conversion, and finally a slightly longer reaction time (8 h) results in >95 % conversion of **2** under relatively mild conditions with low Mn‐corrole loadings.

The dramatic changes observed in the time course UV/Vis spectra for **1 a** during the reaction could be attributed to the effect of axial binding of the oxygen atom of the ring‐opened epoxide, and to the intermediate **B** after CO_2_ insertion/fixation. Finally, we have shown that recycling of the Mn‐corrole is possible and makes the Lewis‐acidic manganese corrole complex reusable for further transformations.

## Conflict of interest

The authors declare no conflict of interest.

## Supporting information

As a service to our authors and readers, this journal provides supporting information supplied by the authors. Such materials are peer reviewed and may be re‐organized for online delivery, but are not copy‐edited or typeset. Technical support issues arising from supporting information (other than missing files) should be addressed to the authors.

Supplementary
