# Supplementary material for: CO2 Fixation with Epoxides under Mild Conditions with a Cooperative Metal Corrole/Quaternary Ammonium Salt Catalyst System
Source: Chem Asian J. 2017 Apr 20;12(10):1048–51. doi: 10.1002/asia.201700354 (PMC5446785; doi:10.1002/asia.201700354)
Supplement: Supplementary file 1 — Supplementary [file ASIA-12-1048-s001.pdf]

CHEMISTRY
AN ASIAN JOURNAL

Supporting Information

CO_2 Fixation with Epoxides under Mild Conditions with a Cooperative Metal Corrole/Quaternary Ammonium Salt Catalyst System

Maximilian Tiffner, Sabrina Gonglach, Michael Haas, Wolfgang Schöfberger,* and Mario Waser*[a]

asia_201700354_sm_miscellaneous_information.pdf

Supplementary information for:

CO_2 Fixation with Epoxides under Mild Conditions with a Cooperative Metal Corrole – Quaternary Ammonium Salt Catalyst System

Maximillian Tiffner[a], Sabrina Gonglach[a], Michael Haas[a], Wolfgang Schöfberger[a,]*, and Mario Waser[a,*]

Institute of Organic Chemistry, Johannes Kepler University,

Altenberger Straße 69, 4040 Linz, Austria.

Email: mario.waser@jku.at, wolfgang.schoefberger@jku.at

Experimental Section

Materials

All chemicals were commercially available and of the highest purity grade and used without further purification. Tetrabutyl ammonium bromide (TBAB) was purchased from Sigma Aldrich. Other used chemicals were purchased either from Sigma Aldrich or Acros chemicals or Alfa Aesar. Reagent grade solvents were purchased from Fischer chemicals and distilled prior to use. THF was distilled over sodium and benzophenone under an argon atmosphere and stored over molecular sieves (4 Å) upon use. TLC was performed by using Fluka silica gel (0.2 mm) on aluminium plates. Silica-gel columns for chromatography were prepared with silica gel 60 (0.060–0.20 mesh ASTM) from Acros.

Instrumentation

Proton (^{1}H NMR) and carbon (^{13}C NMR) spectra were recorded on a Bruker Ascend 700 spectrometer equipped with a cryogenically cooled probe (TXI), or on a Bruker Avance 500 MHz spectrometer. ^{19}F NMR spectra were recorded on a Bruker Avance 300 MHz at 282.4 MHz. The chemical shifts are given in parts per million (ppm) on the delta scale (δ) and are referenced to the residual non-deuterated solvent for ^{1}H and TFA for ^{19}F. MALDI-TOF measurements were collected with a Bruker Autoflex III Smartbeam spectrometer and on an Agilent atmospheric pressure photoionization (APPI) source on an Agilent 6520 quadrupole time-of-flight (QTOF) in the positive mode. UV-Vis absorption spectra were measured on a Varian CARY 100 Bio spectrophotometer.

DFT Calculations

All calculations were performed using the Gaussian 09 package version E02.51. [*1*] Electronic structure calculations were based on Kohn-Sham density functional theory (KS-DFT) with Becke's three-parameter hybrid functional (B3LYP) and a compound basis set, where the Pople's 6-311+G(d,p) basis sets were used for C, H, N, O, F, and Br. For our system, we first performed a tight structural optimization, followed by a frequency calculation to confirm that the optimized structure was indeed a minimum (with no imaginary frequencies). Single-point frequency calculations and NBO analyses were carried out with tight SCF convergence and ultrafine grids in the structural optimizations. Los Alamos National Laboratory 2 (LANL2) relativistic effective core potentials (RECPs) were used to describe the core electrons of Mn atoms. [*2*] The LANL2DZ basis set was augmented by adding one set of polarization functions on all atoms and one set of diffuse functions on all non-hydrogen atoms.

Synthesis Procedures

1) The synthesis of 5,10,15-tris(pentafluorophenyl)corrole (TpFPC) was performed according to [*4*].
2) General procedure for metalation reactions: Free base corroles (0.014 mmol) are dissolved in 3 mL DMF, 34.3 mg (140 µmol, 10 equiv.) manganese(II) acetate tetrahydrate were added and the reaction mixture was refluxed for 30 min under aerobic condition. The DMF was evaporated under reduced pressure and purified *via* column chromatography (silica gel, DCM:MeOH = 2:1). The product **1a** was obtained in 79 % yield.

   Bi^{+III}TpFPC, Sb^{+III}-pyr-TpFPC, $Cu^{+II/+III}$TpFPC, Co^{+III}TpFPC and Co^{+III}-PPh_3-TpFPC, Fe^{+III}-Cl-TpFPC were synthesized according to [*4–11*].

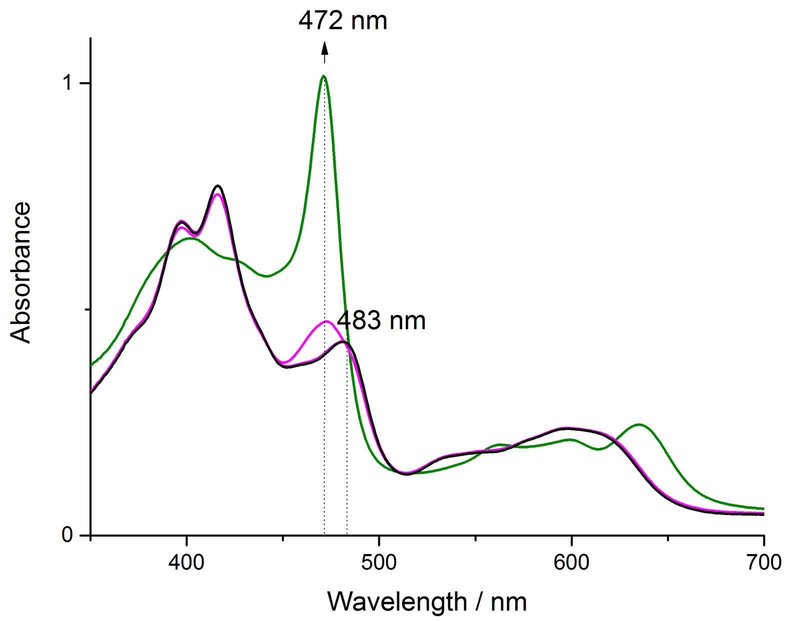

Figure S1: Titration of **1a** (black line) with t-BuOK. Magenta line evolves after addition of stoichiometric amount of t-BuOK; Green line evolves after addition of an excess of t-BuOK.

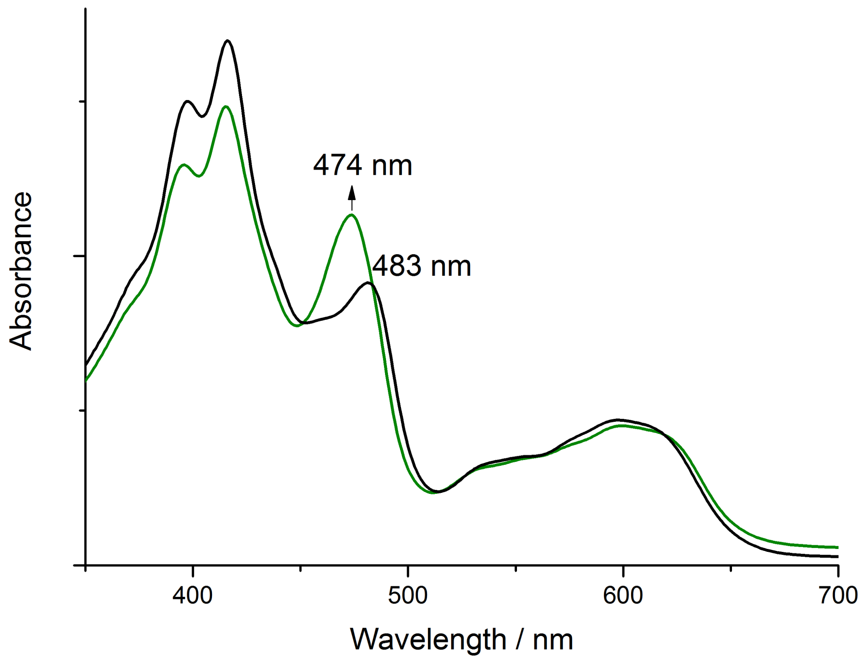

Figure S2: Titration of **1a** (black line) with KOAc (green line).

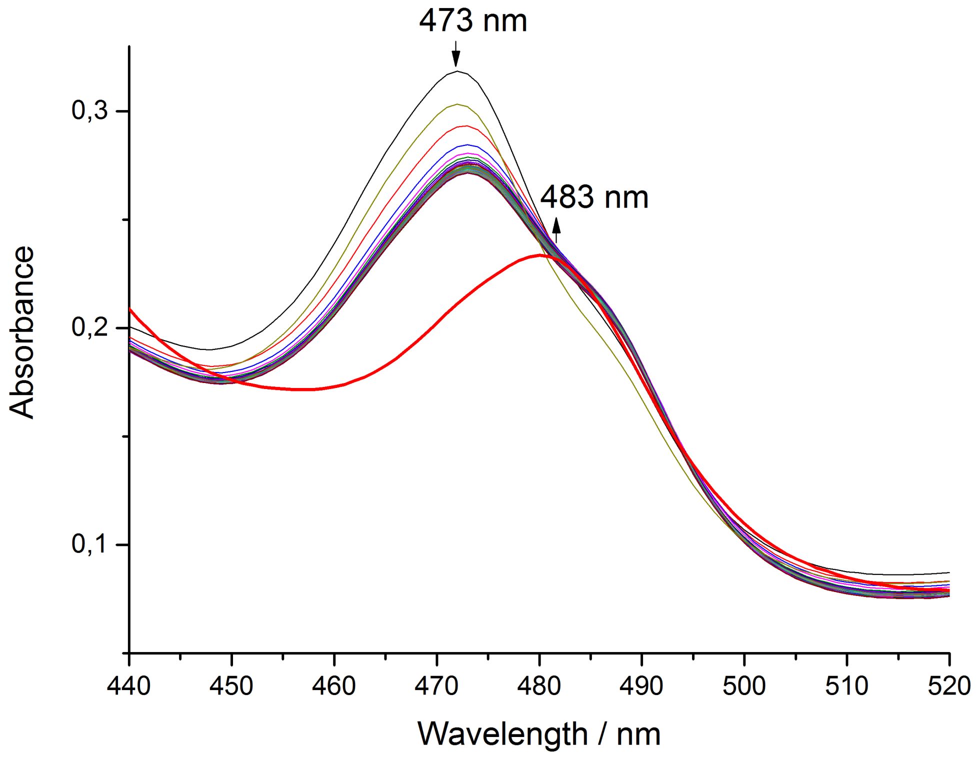

Figure S3: Section of the time-course UV-vis spectra of the back conversion to **1a**.

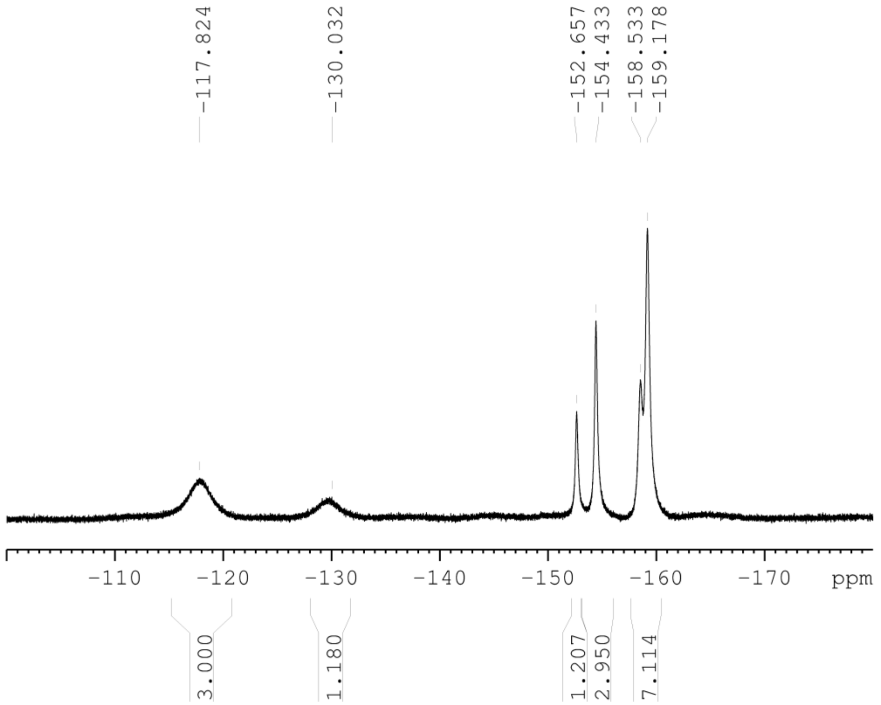

Figure S4: ^{19}F NMR spectrum of the recycled Mn(III)corrole **1a** after the reaction.

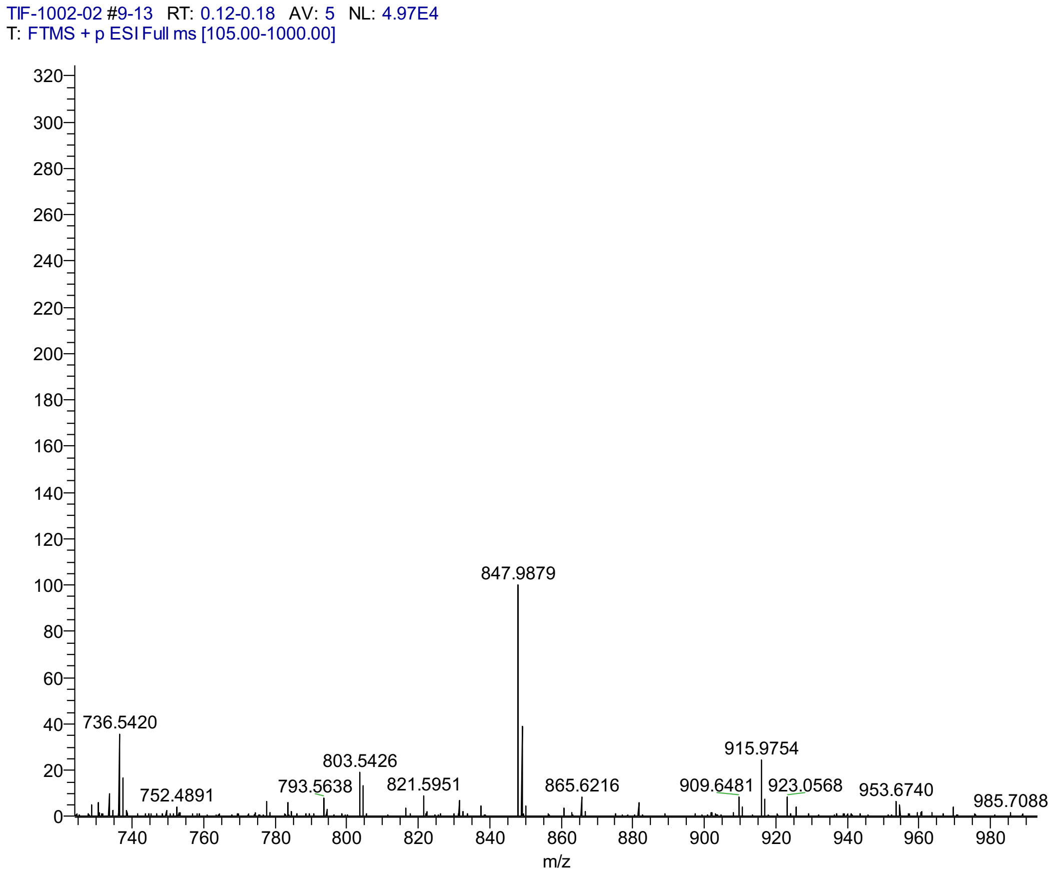

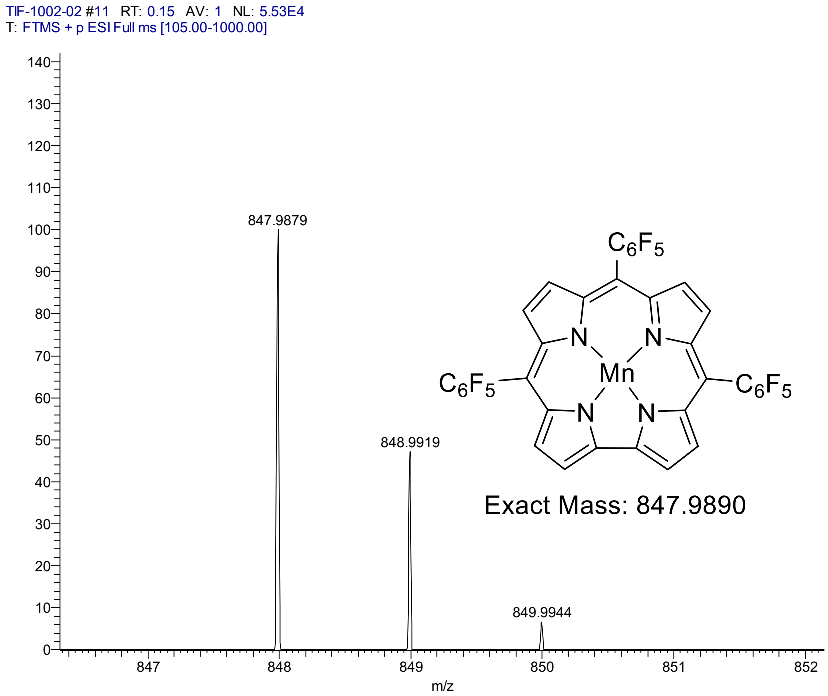

Figure S5: ESI MS spectrum of the recycled Mn(III)corrole **1a** after the reaction.

General experimental procedure for the CO_2 Fixation with Epoxides using a Cooperative Metal Corrole –Quaternary Ammonium Salt Catalyst System:

The reactions were carried out using a Radleys Carousel 12 Plus Reaction Station™. A mixture of 0.002 mmol of the corresponding corrole and 0.08 mmol of Tetra-n-butylammonium bromide (TBAB) was weighted in a reaction tube (ø 16mm). After the addition of 4 mmol epoxide and heating the mixture to 60 °C a CO_2 atmosphere was provided using a balloon and stirring was started (1000 rpm). After removing the balloon and cooling down to room temperature, the crude mixture was directly flushed through a short column of silica gel (heptanes/EtOAc = 10:1–3:1 as eluent) to afford the cyclic carbonates (**S1-9**).

(**S1**) 4-phenyl-1,3-dioxolan-2-one: Isolated Yield: 614 mg (94%). Analytical data are in accordance with those reported in literature. [*12–18*] ^{1}H NMR (300 MHz, δ, $CDCl_3$, 298 K): 7.48-7.39 (m, 3H), 7.38-7.33 (m, 2H), 5.67 (t, J = 8 Hz, 1H), 4.79 (t, J = 8.4 Hz, 1H), 4.43 (dd, J_1 = 8.6 Hz, J_2 = 7.9 Hz, 1H) ppm; ^{13}C NMR (75 MHz, δ, $CDCl_3$, 298 K): 154.7, 135.7, 129.5, 129.0, 125.7, 77.8, 71.0 ppm; HRMS (ESI): m/z calcd for: $C_9H_8NaO_3$ $[M+Na]^+$ 187.0371; found: 187.0367.

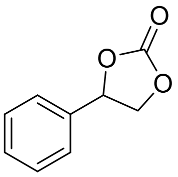

S1

(**S2**) 4-(4-chlorophenyl)-1,3-dioxolan-2-one: Isolated Yield: 759 mg (96%). Analytical data are in accordance with those reported in literature. [*12–18*] ^{1}H NMR (300 MHz, δ, $CDCl_3$, 298 K): 7.42-7.34 (d, 2H, 8.5 Hz), 7.33-7.26 (d, 2H, 8.5 Hz), 5.65 (t, J = 8 Hz, 1H), 4.79 (t, J = 8.5 Hz, 1H), 4.28 (dd, J_1 = 8.6 Hz, J_2 = 7.8 Hz, 1H) ppm; ^{13}C NMR (75 MHz, δ, $CDCl_3$, 298 K): 154.5, 135.4, 134.2, 129.2, 127.2, 77.1, 71.8 ppm; HRMS (ESI): m/z calcd for: $C_9H_7ClNaO_3$ $[M+Na]^+$: 220.9981; found: 220.9978

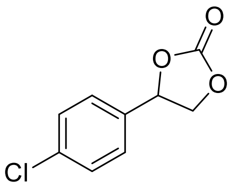

S2

(**S3**) 4-(4-fluorophenyl)-1,3-dioxolan-2-one: Isolated Yield: 711 mg (98%). Analytical data are in accordance with those reported in literature. [*12–18*] ^{1}H NMR (300 MHz, δ, $CDCl_3$, 298 K): 7.41-7.30 (m, 2H), 7.17-7.06 (m, 2H), 5.66 (t, *J* = 8 Hz, 1H), 4.79 (t, *J* = 8.5 Hz, 1H), 4.31 (dd, J_1 = 8.6 Hz, J_2 = 7.8 Hz, 1H) ppm; ^{13}C NMR (75 MHz, δ, $CDCl_3$, 298 K): 163.2 (d *J* = 249 Hz), 154.6, 131.5 (d, *J* = 3.3 Hz), 127.9 (d, *J* = 8.5 Hz), 116.2 (d, *J* = 22,1 Hz), 77.4, 71.0 ppm; 19F NMR (282 MHz, δ, CDCl3, 298 K): -111.1 ppm HRMS (ESI): m/z calcd for: $C_9H_7FNaO_3$ $[M+Na]^+$: 205.0277; found: 205.0271.

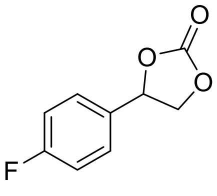

S3

(**S4**) 4-methyl-1,3-dioxolan-2-one: Isolated Yield: 360 mg (88%) when carrying out the reaction at 25 °C for 20 h. Analytical data are in accordance with those reported in literature. [*12–18*] ^{1}H NMR (300 MHz, δ, $CDCl_3$, 298 K): 4.91-4.76 (m, 1H), 4.53 (t, *J* = 7.9 Hz, 1H), 4.00 (t, *J* = 7.8 Hz, 1H), 1.46 (d, *J* = 6.2 Hz, 3H) ppm; ^{13}C NMR (75 MHz, δ, $CDCl_3$, 298 K): 156.1, 73.5, 70.6, 19.2 ppm; HRMS (ESI): m/z calcd for: $C_4H_6NaO_3$ $[M+Na]^+$: 125.0215; found: 125.0207.

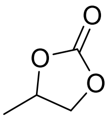

S4

(**S5**) 4-(chloromethyl)-1,3-dioxolan-2-one: Isolated Yield: 475 mg (88%). Analytical data are in accordance with those reported in literature. [*12–18*] ^{1}H NMR (300 MHz, δ, $CDCl_3$, 298 K): 5.04-4.91 (m, 1H), 4.59 (t, *J* = 8.2 Hz, 1H), 4.41 (dd, J_1 = 5.5 Hz, J_2 = 8.0 Hz, 1H), 3.83-3.68 (m, 2H) ppm; ^{13}C NMR (75 MHz, δ, $CDCl_3$, 298 K): 154.3, 74.3, 66.7, 44.1 ppm; HRMS (ESI): m/z calcd for: $C_4H_5ClNaO_3$ $[M+Na]^+$: 158.9825; found: 158.9821.

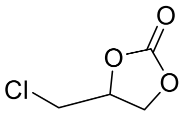

S5

(**S6**) 4-vinyl-1,3-dioxolan-2-one: Isolated Yield: 425 mg (93%). Analytical data are in accordance with those reported in literature. [*12–18*] ^{1}H NMR (300 MHz, δ, $CDCl_3$, 298 K): 4.92-4.78 (m, 1H), 5.50-5.33 (m, 2H), 5.09 (q, *J* = 7.4 Hz, 1H), 4.56 (t, *J* = 8.3 Hz, 1H), 4.10 (t, *J* = 8 Hz, 1H) ppm; ^{13}C NMR (75 MHz, δ, $CDCl_3$, 298 K): 154.7, 132.0, 120.9, 77.2, 68.9 ppm; HRMS (ESI): m/z calcd for: $C_5H_6NaO_3$ $[M+Na]^+$: 137.0215; found: 137.0211.

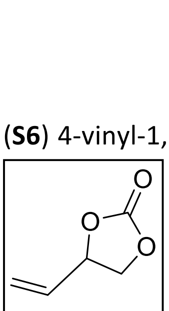

S6

(**S7**) 4-(but-3-en-1-yl)-1,3-dioxolan-2-one: Isolated Yield: 558 mg (98%). Analytical data are in accordance with those reported in literature. [*12–18*] ^{1}H NMR (300 MHz, δ, $CDCl_3$, 298 K): 5.84-5.66 (m, 1H), 5.10-4.96 (m, 2H), 4.77-4.63 (m, 1H), 4.50 (t, *J* = 8.1 Hz, 1H), 4.05 (t, *J* = 7.8 Hz, 1H), 2.32-2.05 (m, 2H), 1.98-1.81 (m, 1H), 1.81-1.65 (m, 1H) ppm; ^{13}C NMR (75 MHz, δ, $CDCl_3$, 298 K): 154.9, 136.0, 116.1, 76.2, 69.2, 32.8, 28.4 ppm; HRMS (ESI): m/z calcd for: $C_7H_{10}NaO_3$ $[M+Na]^+$: 165.0528; found: 165.0526.

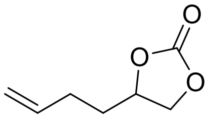

S7

(**S8**) 4-(phenoxymethyl)-1,3-dioxolan-2-one: Isolated Yield: 731 mg (94%). Analytical data are in accordance with those reported in literature. [*12–18*] ^{1}H NMR (300 MHz, δ, $CDCl_3$, 298 K): 7.31 (t, *J* = 7.8 Hz, 2H), 7.02 (t, *J* = 7.4 Hz, 1H), 6.91 (d, *J* = 8.1 Hz), 5.09-4.96 (m, 1H), 4.60 (t, *J* = 8.5 Hz, 1H), 4.51 (dd, J_1 = 6.0 Hz, J_2 = 8.4 Hz, 1H), 4.24 (dd, J_1 = 10.5 Hz, J_2 = 3.8 Hz, 1H),), 4.12 (dd, J_1 = 10.5 Hz, J_2 = 3.8 Hz, 1H) ppm; ^{13}C NMR (75 MHz, δ, $CDCl_3$, 298 K): 157.7, 154.7, 129.5, 121.8, 114.5, 74.1, 66.7, 66.1 ppm; HRMS (ESI): m/z calcd for: $C_{10}H_{10}NaO_4$ $[M+Na]^+$: 217.0477; found: 217.0475.

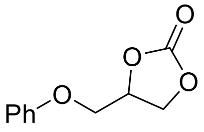

S8

(**S9**) 4-((benzhydryloxy)methyl)-1,3-dioxolan-2-on: Isolated Yield: 810 mg (70%). Analytical data are in accordance with those reported in literature. [*12–18*] ^{1}H NMR (300 MHz, δ, $CDCl_3$, 298 K): 7.38-7.23 (m, 10H), 5.42 (s, 1H),4.79-4.70 (m, 1H), 4.43-4.31 (m, 2H), 3.68 (dd, J_1 = 11.0 Hz, J_2 = 3.3 Hz, 1H), 4.24 (dd, J_1 = 11.0 Hz, J_2 = 3.4 Hz, 1H), ppm; ^{13}C NMR (75 MHz, δ, $CDCl_3$, 298 K): 154.9, 141.0, 140.9, 128.3, 128.2, 127.6, 127.5, 126.6, 126.5, 83.8, 74.9, 67.6, 66.0 ppm; MS (ESI): m/z calcd for: $C_{17}H_{20}NO_4$ $[M+NH_4]^+$: 302.1; found: 302.1.

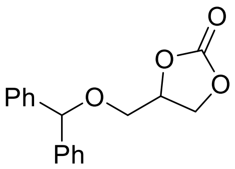

S9

References

1. Frisch MJ, Trucks GW, Schlegel HB, Scuseria GE, Robb MA, Cheeseman JR, Scalmani G, Barone V, Mennucci B, Petersson GA, Nakatsuji H, Caricato M, Li X, Hratchian HP, Izmaylov AF, Bloino J, Zheng G, Sonnenberg JL, Hada M, Ehara M, Toyota K, Fukuda R, Hasegawa J, Ishida M, Nakajima T, Honda Y, Kitao O, Nakai H, Vreven T, Montgomery JA, Peralta JE, Ogliaro F, Bearpark M, Heyd JJ, Brothers E, Kudin KN, Staroverov VN, Kobayashi R, Normand J, Raghavachari K, Rendell A, Burant JC, Iyengar SS, Tomasi J, Cossi M, Rega N, Millam JM, Klene M, Knox JE, Cross JB, Bakken V, Adamo C, Jaramillo J, Gomperts R, Stratmann RE, Yazyev O, Austin AJ, Cammi R, Pomelli C, Ochterski JW, Martin RL, Morokuma K, Zakrzewski VG, Voth GA, Salvador P, Dannenberg JJ, Dapprich S, Daniels AD, Farkas, Foresman JB, Ortiz JV, Cioslowski J and Fox DJ. *Gaussian 09, Revision B.01, Gaussian, Inc., Wallingford CT* 2009;
2. P. Jeffrey Hay and Willard R. Wadt. *J. Chem. Phys* 1985; **82**: 299–310.
3. Mark E. Casida, Christine Jamorski, Kim C. Casida and Dennis R. Salahub. *J. Chem. Phys* 1998; **108**: 4439–4449.
4. Reith LM, Stiftinger M, Monkowius U, Knör G and Schoefberger W. *Inorg. Chem.* 2011; **50**: 6788–6797.
5. Luobeznova I, Raizman M, Goldberg I and Gross Z. *Inorg. Chem.* 2006; **45**: 386–394.
6. König M, Reith LM, Monkowius U, Knör G, Bretterbauer K and Schoefberger W. *Tetrahedron* 2011; **67**: 4243–4252.
7. Lei H, Fang H, Han Y, Lai W, Fu X and Cao R. *ACS Catal* 2015; **5**: 5145–5153.
8. Kadish KM, Shen J, Frémond L, Chen P, Ojaimi ME, Chkounda M, Gros CP, Barbe J, Ohkubo K, Fukuzumi S and Guilard R. *Inorg. Chem.* 2008; **47**: 6726–6737.
9. Chen C, Zhu Y, Fan Q, Song H and Zheng J. *Tetrahedron Lett.* 2013; **54**: 4143–4147.
10. Simkhovich L, Mahammed A, Goldberg I and Gross Z. *Chem. Eur. J.* 2001; **7**: 1041–1055.
11. Simkhovich L, Goldberg I and Gross Z. *Inorg. Chem.* 2002; **41**: 5433–5439.
12. Rokicki G, Kuran W and Pogorzelska-Marciniak B. *Monatsh. Chem.* 1984; **115**: 205–214.
13. Corsano S, Strappaghetti G, Ferrini R and Sala R. *Arch. Pharm.* 1988; **321**: 731–734.
14. Clegg W, Harrington RW, North M and Pasquale R. *Chem. Eur. J.* 2010; **16**: 6828–6843.
15. Wu L, Yang H, Wang H and Lu J. *RSC Adv* 2015; **5**: 23189–23192.
16. Büttner H, Steinbauer J and Werner T. *ChemSusChem* 2015; **8**: 2655–2669.
17. Babu R, Roshan R, Kathalikkattil AC, Kim DW and Park D. *ACS Appl. Mater. Interfaces* 2016; **8**: 33723–33731.
18. Shen Y, Duan W and Shi M. *Eur. J. Org. Chem.* 2004; **2004**: 3080–3089.
